# Properties of the Sodium Naproxen-Lactose-Tetrahydrate Co-Crystal upon Processing and Storage

**DOI:** 10.3390/molecules21040509

**Published:** 2016-04-19

**Authors:** Ioana Sovago, Wenbo Wang, Danwen Qiu, Dhara Raijada, Jukka Rantanen, Holger Grohganz, Thomas Rades, Andrew D. Bond, Korbinian Löbmann

**Affiliations:** 1 Department of Pharmacy, University of Copenhagen, Copenhagen 2100, Denmark; ioana.sovago@chem.ku.dk (I.S.); wwbsgdtc@126.com (W.W.); danwenqiu@live.com (D.Q.); dhara.niper@gmail.com (D.R.); jukka.rantanen@sund.ku.dk (J.R.); holger.grohganz@sund.ku.dk (H.G.); thomas.rades@sund.ku.dk (T.R.); 2 Department of Chemistry, University of Cambridge, Cambridge CB2 1EW, UK; adb29@cam.ac.uk

**Keywords:** co-crystal 1, co-amorphous 2, dehydration 3

## Abstract

Co-crystals and co-amorphous systems are two strategies to improve the physical properties of an active pharmaceutical ingredient and, thus, have recently gained considerable interest both in academia and the pharmaceutical industry. In this study, the behavior of the recently identified sodium naproxen-lactose-tetrahydrate co-crystal and the co-amorphous mixture of sodium, naproxen, and lactose was investigated. The structure of the co-crystal is described using single-crystal X-ray diffraction. The structural analysis revealed a monoclinic lattice, space group *P*2_1,_ with the asymmetric unit containing one molecule of lactose, one of naproxen, sodium, and four water molecules. Upon heating, it was observed that the co-crystal transforms into a co-amorphous system due to the loss of its crystalline bound water. Dehydration and co-amorphization were studied using synchrotron X-ray radiation and thermogravimetric analysis (TGA). Subsequently, different processing techniques (ball milling, spray drying, and dehydration) were used to prepare the co-amorphous mixture of sodium, naproxen, and lactose. X-ray powder diffraction (XRPD) revealed the amorphous nature of the mixtures after preparation. Differential scanning calorimetry (DSC) analysis showed that the blends were single-phase co-amorphous systems as indicated by a single glass transition temperature. The samples were subsequently tested for physical stability under dry (silica gel at 25 and 40 °C) and humid conditions (25 °C/75% RH). The co-amorphous samples stored at 25 °C/75% RH quickly recrystallized into the co-crystalline state. On the other hand, the samples stored under dry conditions remained physically stable after five months of storage, except the ball milled sample stored at 40 °C which showed signs of recrystallization. Under these dry conditions, however, the ball-milled co-amorphous blend crystallized into the individual crystalline components.

## 1. Introduction

Co-crystals and co-amorphous systems have in the past few years attracted considerable interest in the pharmaceutical field because they potentially possess different physical properties compared to the individual drugs in different polymorphic, hydrate, or pure amorphous forms [1,2]. A number of potential improvements with these two formulation strategies have been reported, including improved bioavailability through increasing solubility and dissolution, increased physical and chemical stability, improved flowability, and tabletability, and favorable water-solid interactions [2,3,4,5]. Finding the optimal solid form of an active pharmaceutical ingredient is of major clinical relevance and also provides important intellectual property opportunities for the pharmaceutical industry [6,7]. In this context, co-crystals and co-amorphous forms are of high interest as they allow to prolong market exclusivity of a certain drug through life cycle management by re-formulating the drug in a different solid form [2].

Both co-crystals and co-amorphous drug formulations are single-phase, solid, multi-component mixtures, where the drug forms supramolecular assemblies with one or more components, often involving hydrogen bonding between the components in the mixture [1,6,8]. They are subcategories of solid dispersions as defined by Chiou and Riegelman [9]. Accordingly, solid dispersions are multicomponent mixtures and differ in the number of solid-state phases and the physical state thereof. They are classified into four groups: eutectic mixtures (two phases, both crystalline), solid solution (one phase, crystalline), glass suspension (two phases, both amorphous), and glass solution (one phase, amorphous). Within this classification co-crystals fall into the group of solid solutions, whereas co-amorphous systems fall into the group of glass solutions.

As the name implies, a co-crystal is crystalline in nature. Where there are hydrogen bonds, the absence of hydrogen transfer distinguishes them from salts [10]. In contrast, co-amorphous systems do not possess a three-dimensional long-range order of the components in the mixture; however, short-range order has been reported in the form of hetero-dimers between the components in binary co-amorphous systems [11,12,13]. Seemingly, both systems appear to be in close relation to each other. Both co-crystals and co-amorphous systems have been prepared in stoichiometric ratios; and they may be stabilized through the same hydrogen-bonding motifs. However, to our knowledge there have been no reports on the transformation from co-crystal to co-amorphous and *vice versa*. Upon storage of co-amorphous systems, for example, it has been frequently reported that the components of co-amorphous mixtures undergo a phase separation and crystallize back into the individual components [5,11,14,15]. On the other hand, it has been suggested that the individual amorphous forms of the components can lead to molecular association of the molecules and subsequently to the formation of a co-crystal upon heating [16]. Furthermore, a relation between hydrogen bonding capacities of the co-former on the co-crystal/co-amorphous formation upon milling has been reported [15]. The authors found that sulfathiazole formed co-amorphous mixtures with different carboxylic acids when the hydrogen bonding capacities of the co-former exceeded those of sulfathiazole. However, when the hydrogen bonding capacities where similar to sulfathiazole, the milling of equimolar ratios between drug and co-former resulted either in the formation of a co-crystal, salt, or the components remained individually crystalline.

Sodium naproxen has been shown to exhibit different hydration states including a monohydrate, two dihydrate forms and a tetrahydrate [17,18,19,20]. The tetrahydrated form of sodium naproxen was identified to be the most soluble at different pH conditions [21]. However, the stability of the tetrahydrate form is rather poor at ambient conditions showing rapid dehydration and additional excipients are required to stabilize this form. For instance, as it was recently shown, sodium naproxen forms tetrahydrated co-crystals with lactose (NaN-Lact-4H_2_O) [21]. As mentioned above, understanding the behavior of the drugs in different hydrated states or with additional excipients is extremely important in the pharmaceutical field. One step in this direction consists of linking the drug properties with the molecular structure in the solid state.

In this study, the crystal structure of the recently identified NaN-Lact-4H_2_O co-crystal will be described and its dehydration behavior during thermal treatment will be investigated. The appearance of a co-amorphous NaN-Lact upon dehydration will subsequently be studied and compared to other co-amorphous preparative techniques, including spray drying and ball milling. Furthermore, the behavior of the differently prepared co-amorphous systems will be tested under different storage conditions.

## 2. Results and Discussion

### 2.1. Molecular Structure of the Na-Naproxen Lactose Tetrahydrate (NaN-Lact-4H_2_O) Co-Crystal

**Figure 1:**
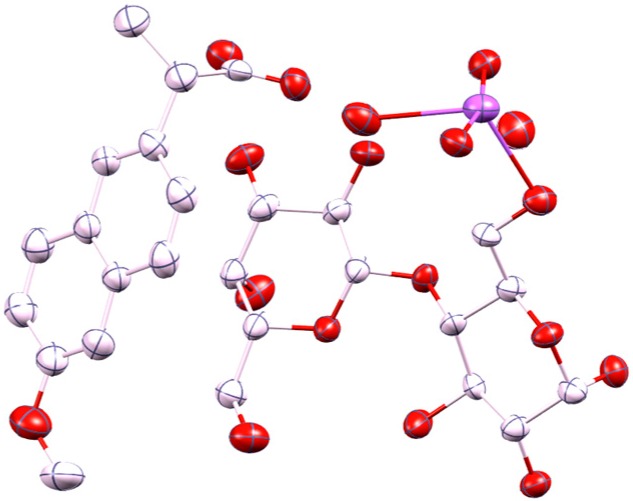
Displacement ellipsoid plot of the NaN-Lact-4H_2_O co-crystal showing the asymmetric unit (ellipsoids drawn at the 50% probability level); color code: Na-purple, O-red, C-light pink; H atoms are omitted.

**Figure 2:**
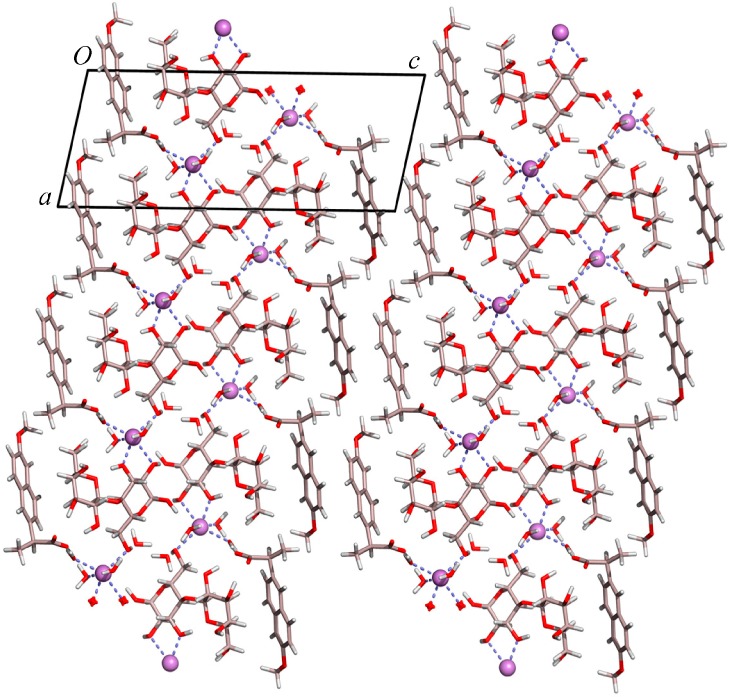
The crystal packing of NaN-Lact-4H_2_O, showing the water molecules included in the chains along the *b* axis; color code: Na-purple, O-red, C-light pink, H-white.

**Figure 3:**
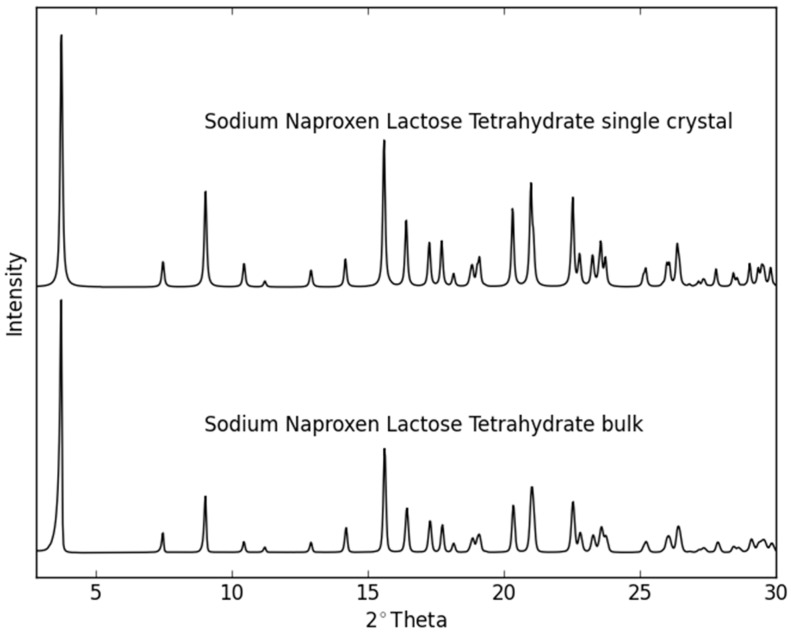
The powder diffraction pattern simulated from the NaN-Lact-4H_2_O co-crystal structure and the experimentally-determined XRPD diffractogram of the bulk material.

Single crystal X-ray diffraction data at 21(2) °C revealed a monoclinic lattice: *a =* 9.9900(6), *b* = 6.4720(4), *c* = 24.1480(17), and β = 101.632(4)°. The system crystallized in space group *P*2_1_ with the asymmetric unit comprising one molecule of lactose, one of naproxen, four water molecules, and one sodium ion. Therefore, the crystal structure determination confirms the presence of the four water molecules within the formula unit (Figure 1), as previously determined using TGA measurements [21]. The Na^+^ ion is hexacoordinated by three water molecules and three oxygen atoms of the lactose. The fourth water molecule lies independent forming chains along the *b* axis and linking the lactose molecules via H bonding interactions within the crystal packing (Figure 2). Furthermore, the molecules alternate forming layers of naproxen followed by lactose, Na^+^ ion, water, Na^+^ ion, lactose, and naproxen. The units repeat along the *c* axis and are connected via H bond interactions. The powder pattern of the previously identified NaN-Lact-4H_2_O co-crystals showed identical features with the simulated powder pattern of the determined co-crystal structure (Figure 3).

### 2.2. Dehydration Behavior of the NaN-Lact-4H_2_O Co-Crystal and Co-Amorphization

It has previously been shown that NaN-Lact-4H_2_O transformed into an amorphous form during heating [21]. TGA measurements revealed a weight loss of 10.1% in the 60–120 °C range corresponding to approx. four water molecules present in the co-crystal. As described above, the water molecules in the co-crystal are bound in different ways, *i.e.*, either coordinated to the Na^+^ ion (75%) or in the chains along the *b* axis forming channels (25%). Interestingly, although the water molecules are placed quite differently within the crystal packing, only one dehydration step was observed in the TGA thermograms described by Raijada *et al.* [21]. In contrast, previous reports on sodium naproxen dehydrate [20] showed two dehydration steps, even though all water molecules are coordinated to the Na^+^ ion. In that case, the dehydration passes through a monohydrate phase before forming the sodium naproxen anhydrate form. In the case of the sodium naproxen dihydrate, the Na^+^ ion is directly coordinated by naproxen molecules that form layers within the crystal packing, so that they can rearrange after the loss of one water molecule.

**Figure 4:**
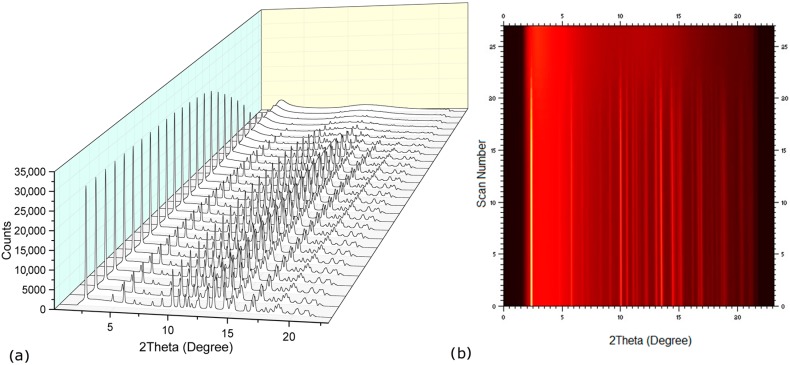
Synchrotron powder data collected upon heating from 27 to 127 °C, showing the transformation from the NaN-Lact-4H_2_O co-crystalline state to the NaN-Lact co-amorphous form. (**a**) powder patterns, (**b**) view of the intensities.

In order to investigate the dehydration process and formation of other possible hydrate/solid forms, synchrotron powder X-ray diffraction data were collected during heating above 120 °C. The diffraction patterns (Figure 4) showed a direct transformation of the tetrahydrate co-crystals into a co-amorphous form. Remarkably, all water molecules appear to leave the co-crystal simultaneously, regardless of their different association in the crystal lattice. According to Galwey [22], the dehydration of NaN-Lact-4H_2_O appears to follow a water evolution type 3 (WET 3), where the mechanism of water loss is driven by structural changes at the interface and the structure loss upon dehydration results in an amorphous product. This may be a confirmation that the water molecules play a key role in holding the sodium naproxen and lactose molecules together via hydrogen bond interactions within the crystal packing. Upon heating, the water molecules are leaving the crystal; consequently the ordered sodium naproxen and lactose molecules collapse into an amorphous form. A rearrangement of the molecules is not affordable in this case. A direct transition from a hydrated co-crystal to a co-amorphous form upon heating is an interesting finding as it may offer the possibility to prepare co-amorphous systems in an easy and scalable manner and, thus, be a competitive alternative to other frequently used techniques to prepare co-amorphous forms such as ball milling. Similarly, dehydration has also been suggested as a technique for the preparation of individual amorphous materials such as the dehydration of carbamazepine dihydrate upon heating [23].

**Figure 5:**
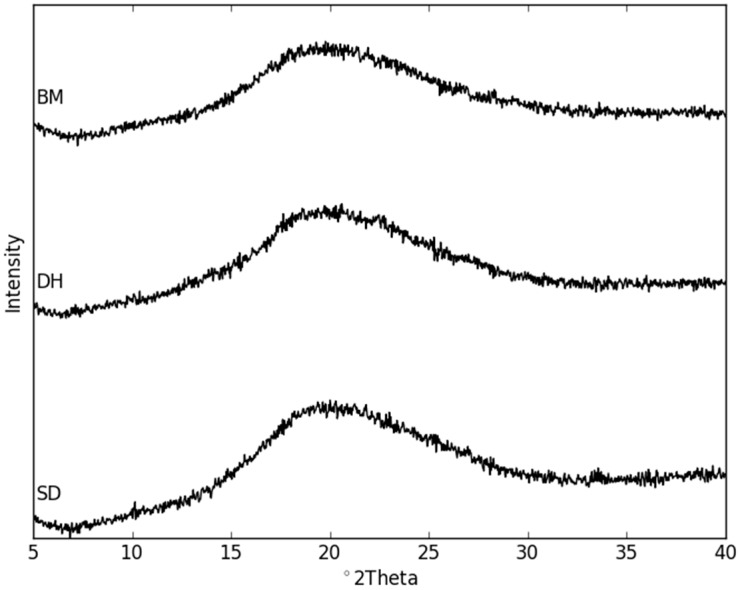
X-ray powder diffraction patterns of the freshly prepared co-amorphous BM, DH, and SD samples.

Some previous studies have shown that the preparation method and processing parameters can have a significant impact on the physical stability of amorphous drugs [24,25]. Subsequently, different processing techniques, *i.e.*, ball milling (BM), spray drying (SD), and co-crystal dehydration (DH), were used to prepare the co-amorphous mixture of NaN and Lact. XRPD measurements revealed that all preparation methods resulted in amorphization of the mixtures as indicated by the appearance of a “halo” in the diffractograms (Figure 5). Furthermore, DSC analysis showed that the blends were homogeneous single-phase co-amorphous systems as indicated by a single glass transition temperature. The highest T_g_ was observed for the DH samples (85.3 ± 1.0 °C), followed by the BM (74.4 ± 0.8 °C) and SD samples (66.3 ± 1.1 °C). Interestingly, the T_g_ values of the differently prepared co-amorphous NaN-Lact samples were in a very narrow range within the same preparation method, however, they were very different amongst the different techniques.

**Figure 6:**
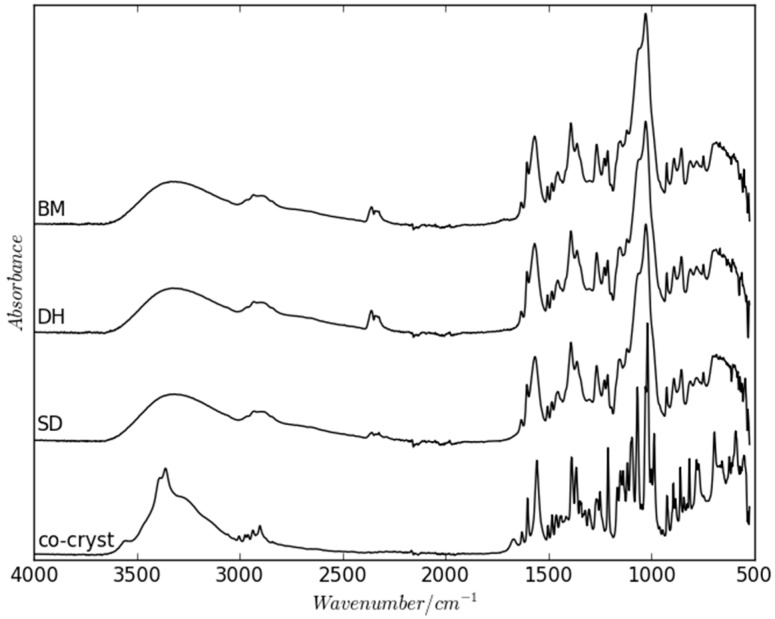
FT–IR spectra of the freshly prepared co-amorphous BM, DH and SD samples, as well as the NaN-Lact-4H_2_O co-crystal.

The physical properties of pure amorphous materials can differ when prepared with different methods and is a well-known phenomenon. Pure amorphous indomethacin, for example, was found to show measurable differences at the molecular level depending on its preparative method that resulted in T_g_ differences of up to 5 °C [25]. Differences in T_g_ have also been reported for amorphous multi-component systems, such as co-amorphous indomethacin and arginine prepared by either ball milling or spray drying [26]. However, FT–IR analysis of the differently prepared co-amorphous NaN-Lact samples revealed that the arrangement on the molecular level appears to be very similar in all samples with only small changes that can be observed (Figure 6).

Another possibility for the detected differences in the T_g_ values, depending on the preparative technique used, could be differences in residual or absorbed moisture, since amorphous materials generally are hygroscopic [27]. However, TGA revealed similar water content for all samples, *i.e.*, DH (3.2% ± 0.5%), BM (3.9% ± 0.5%), and SD (2.2% ± 0.9%). Furthermore, the temperature range for dehydration (25–100 °C) of all materials upon heating was very similar, indicating that the absorbed water was bound in a similar way regardless of the preparation method. Overall, the results of TGA suggested that the observed differences in T_g_ are not a result of differences in the residual moisture content of the samples. It is also worth mentioning that the overall residual water content of the co-amorphous materials (~3%) was much lower than the crystalline water content of 10.1% found in the co-crystal.

### 2.3. Physical Stability of Co-Amorphous NaN-Lact

**Figure 7:**
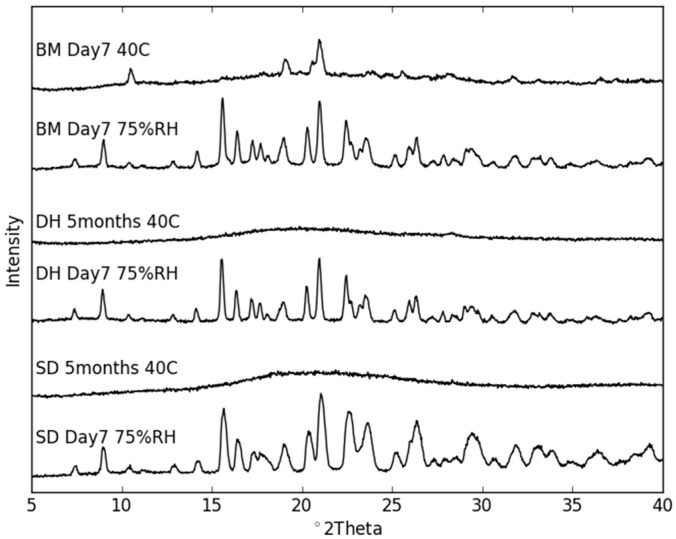
XRPD diffractograms of co-amorphous BM, DH and SD samples stored for seven days (BM) and five months (DH and SD) at 40 °C under dry conditions, as well as for seven days at 25 °C/75% RH.

**Figure 8:**
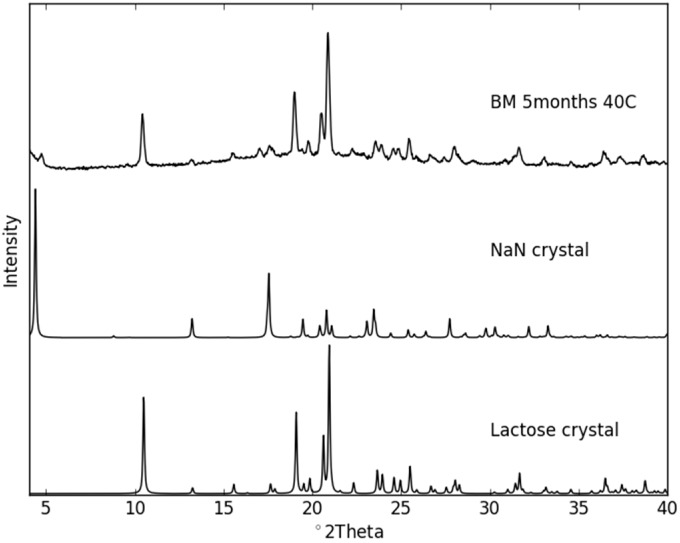
Recrystallization XRPD pattern of ball milled co-amorphous NaN-Lact after five months storage at 40 °C, showing the predominant crystalline powder pattern of Lact and a small contribution of crystalline NaN. NaN crystal and Lact crystal diffraction patterns were simulated from the known crystal structures (CSD refcodes: ASUBUL for NaN and BLACTO for Lact).

The co-amorphous samples were stored at different conditions (Table 1) for up to five months and periodically analyzed towards recrystallization using XRPD and FT–IR. As expected, the samples stored under dry conditions were markedly more stable than those stored at humid conditions (Figure 7). All samples stored at 25 °C/75% RH rapidly recrystallized within seven days. A comparison of the XRPD patterns revealed that the samples crystallized into the NaN-Lact-4H_2_O co-crystal in the presence of moisture. On the other hand, all samples stored dry at 25 °C remained co-amorphous over the entire stability study (data not shown). However, at 40 °C under dry conditions, the co-amorphous samples prepared in different ways also showed differences in their tendency to recrystallize. While the SD and DH samples remained co-amorphous even after five months, the BM samples revealed diffraction peaks in the XRPD patterns already after seven days (Figure 7). The crystalline sample composition of the recrystallization product of the BM samples comprises the original crystalline structures of both NaN and Lact. As shown in Figure 8, the predominant crystalline peaks are given by Lact with a small contribution of NaN. For instance there is a small intensity peak around 4.3° 2θ that can be attributed to NaN. Interestingly, the T_g_ of the BM samples was higher than that of the SD samples. Nevertheless, the BM samples were less stable than the SD samples. On the other hand, the BM samples contained more residual moisture compared to the SD and DH samples (see Section 2.2.). Since water is a potent plasticizer, the larger amount of absorbed moisture may have contributed to the lower stability of the BM samples. With respect to the preparative techniques BM and SD, the findings for physical stability of co-amorphous systems are in line with previously reported findings on individual amorphous samples [25].

## 3. Materials and Methods

### 3.1. Materials

**Figure 9:**
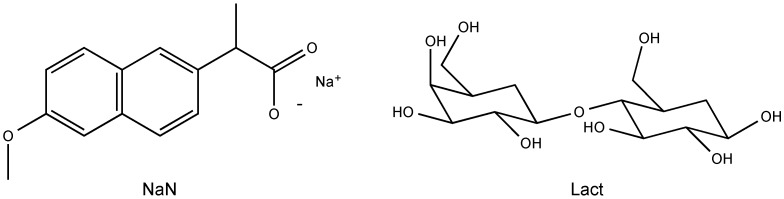
Chemical structures of sodium naproxen (NaN) and lactose (Lact).

Sodium naproxen anhydrate (NaN) was received from Divi’s Laboratories Limited (Hyderabad, India). Anhydrous lactose (Lact) (Supertab^®^ 22AN) was received from DMV-Fonterra Excipients (Goch, Germany). All materials were of analytical grade and used as received. The chemical structures of both materials are shown in Figure 9.

### 3.2. Methods

#### 3.2.1. Preparation of the (NaN-Lact-4H_2_O) Co-Crystal

NaN-Lact tetrahydrate (NaN-Lact-4H_2_O) co-crystals were prepared as bulk material as previously described [19]. Briefly, NaN and Lact were dissolved in warm water at approx. 50 °C under constant stirring for 30 min. The solution was allowed to evaporate slowly under ambient conditions to give needle-shaped crystals within one week.

#### 3.2.2. Preparation of the NaN-Lact Co-Amorphous System

The co-amorphous NaN-Lact at a 1:1 molar ratio was prepared using three different methods: spray drying, ball milling, and dehydration of the NaN-Lact-4H_2_O co-crystal.

##### Spray Drying (SD)

NaN (2.71 g)—Lact (2 g) were dissolved in 100 mL MilliQ water and spray dried using a Büchi B-290 spray dryer (Büchi Labortechnik AG, Flawil, Switzerland). The following processing conditions were applied: inlet temperature: 150 °C, resulting outlet temperature: 63–65 °C, aspirator: 95%, feed rate: 3 mL/min, drying N_2_ flow: 40 m^3^/h.

##### Ball Milling (BM)

NaN-Lact (total mass of 500 mg) at a 1:1 molar ratio was milled using an oscillatory ball mill (MixerMill MM400, Retsch GmbH and Co., Haan, Germany) at 30 Hz frequency for 90 min. The samples were placed in 25 mL stainless steel containers and milled using stainless steel balls (12 mm) in a cold room at 6 °C.

##### Dehydration of the Co-Crystal (DH)

NaN-Lact-4H_2_O co-crystalline powder (~2 g) was kept isothermally at 110 °C in an oven for 30 min for a complete removal of crystalline water and transformation from a co-crystalline state into a co-amorphous form.

#### 3.2.3. Analytical Techniques

##### Single-Crystal X-ray Diffraction and Crystal Structure Determination

Single-crystal X-ray diffraction data of NaN-Lact-4H_2_O were collected at 21 °C on a Bruker D8-QUEST diffractometer (Bruker AXS: Madison, Wisconsin, WI, USA) with an IμS Cu microsource (λ = 1.5418 Å) and a PHOTON-100 detector. The obtained images were integrated and processed using *SAINT* within *APEX2* (*APEX2 software*; Bruker AXS Inc.: Madison, Wisconsin, WI, USA, 2012). Crystal structure solution and refinements were performed using SHELXL-2013 [28] with full-matrix least-squares on *F*^2^ and including all of the unique data. Anisotropic displacement parameters were refined for non-H atoms. The H atoms bonded to O atoms were visible in difference Fourier maps for only some of the O atoms. H atoms on the water molecules were not clearly visible. Therefore, the H atoms were placed so as to form a reasonable H-bond network. In a following step, the structure was energy minimized using DFT-D methods (*CASTEP*) [29], with only the H atoms allowed to move (non-H atoms and unit cell fixed). The resulting H atom positions were transferred to the model and then allowed to ride on their parent atoms for the final cycles of refinement. The H-atom positions in the final model are, thus, closer to the neutron positions than X-ray positions. Subsequent energy minimization of this structure with all atoms allowed to move within a constrained unit cell, then also with the unit cell relaxed, produced negligible deviations, thereby confirming that the model represents a viable energy minimum. The crystal structure has been deposited in the Cambridge Structural Database (CSD) [30] with deposition number CCDC 1042938. The applied experimental details are listed in Table 2.

##### X-ray Powder Diffraction (XRPD) Measurements

Laboratory Measurements: XRPD data were collected using a X’Pert Pro X-ray diffractometer (PANalytical, Almelo, The Netherlands; MPD PW3040/60 XRD; non-monochromated Cu Kα radiation; λ = 1.5418 Å; 45 kV; 40 mA). The samples were measured using reflection mode over a range of 2°–40° 2θ with a point resolution of 0.026° 2θ and a total collection time of 10 min.

Synchrotron Measurements: The behavior of NaN-Lact-4H_2_O upon heating was investigated at Beamline I711, MAXLab (Lund, Sweden), using an Agilent Titan CCD area detector, with λ = 0.9934 Å. The CCD images were integrated using *Fit2D* (Fit2D software, Andy Hammersley, ESRF, 2004). The 2θ range achieved after integration was 2.2°–21.0° (*d* ≈ 25.9–2.7 Å) with an effective angular step size of 0.016° 2θ. Samples were loaded in glass capillaries with an outside diameter of 1 mm, and ramped from 27 to 127 °C with a heating rate of approx. 11 K/min using an N_2_ cryostat (Oxford Instruments Cryojet, Oxford Instruments, Abingdon, UK). A total of 27 diffraction images were recorded at 10 s per image.

##### Modulated Differential Scanning Calorimetry (mDSC)

mDSC analysis was performed in modulated temperature mode using a TA-Discovery DSC (TA instruments, New Castle, DE, USA) under a constant nitrogen flow rate of 50 mL/min. The samples (approx. 5 mg) were crimped into aluminum Tzero pans and lids and heated to 150 °C at a heating rate of 2 °C/min with a modulation amplitude of 0.212 °C and a modulation period of 40 s. The glass transition temperatures (T_g_) were determined from the resulting thermograms as a midpoint of the onset and endset temperature in the reversing heat flow signal (*n* = 3).

##### Thermogravimetric Analysis (TGA)

The samples were measured using a TA-Discovery TGA (TA instruments). Approximately 10 mg of samples were placed into platinum pans and heated from room temperature to 500 °C at a heating rate of 10 °C/min. Water content was calculated as weight loss between room temperature and 140 °C using the resulting weight-temperature thermograms.

##### FT-Infrared Spectroscopy (FT–IR)

FT–IR spectra were recorded using a Nicolet 380 FT–IR (Thermo Scientific, Madison, WI, USA) attached with an attenuated total reflectance accessory (ATR, Smart iTR, Thermo Scientific). Spectra were collected over a range of 4000–400 cm^−1^ (64 scans, resolution 4 cm^−1^).

##### Stability Studies

The samples obtained by different preparation techniques were kept under different storage conditions, as summarized in Table 1. Dry conditions were obtained by storing the samples over silica gel. A relative humidity of 75% RH was obtained by a saturated NaCl solution. The stored samples were tested towards recrystallization by XRPD and FTIR at predetermined time points (day 7, 14, 28, 41, 61, and after five months)

## 4. Conclusions

In this study, the transformation of the NaN-Lact-4H_2_O co-crystal into a co-amorphous NaN-Lact form and its recrystallization behavior upon storage were investigated. The X-ray crystal structure of the NaN-Lact-4H_2_O revealed different environments for the water molecules within the crystal packing. Nevertheless, the dehydration upon heating was found to occur in a single step and resulted in a co-amorphous product. Subsequently, three different preparation routes were used to prepare co-amorphous NaN-Lact, and solid-state differences between the samples were reflected in differences in their glass transition temperatures. However, the samples appeared to be similar on their molecular level arrangement and with respect to residual moisture. Storing the differently- prepared co-amorphous samples at a high relative humidity (75%) quickly transformed the entire samples back into the NaN-Lact-4H_2_O co-crystalline state within seven days. Under dry conditions, all samples remained amorphous over a period of five months at 25 °C, however, at elevated temperature (40 °C), only the co-amorphous blends prepared by BM crystallized into the individual crystalline components, whereas the SD and DH samples remained amorphous. Therefore, the preparative technique can have a measureable impact on the performance of co-amorphous systems and suggests that a suitable method of production for co-amorphous drug formulation needs to be investigated and carefully selected.

## Figures and Tables

**Table 1:** Different storage conditions of sodium naproxen-lactose systems.

Condition	Temperature (°C)	Humidity (% RH)
Condition 1	25	<5
Condition 2	25	75
Condition 3	40	<5

**Table 2:** Experimental details.

Compound Formula	C_26_H_43_NaO_18_
*M_r_*	666.59
Space group	*P*2_1_
Crystal system	monoclinic
*a*/Å	9.9900(6)
*b*/Å	6.4720(4)
*c*/Å	24.1480(17)
β/°	101.632(4)
*V/*Å^3^	1529.23(17)
*Z*	2
*D_calc_*/g·cm^−3^	1.448
λ/Å	1.5418
μ/mm^−1^	1.174
Temperature/°C	21(2)
Crystal size/mm	0.20 × 0.05 × 0.05
θ range/°	7.804–54.237
Max sin(θ)/λ	0.52
No. of unique data	3728
*hkl* range	−10 ≤ *h* ≤ 10
	−6 ≤ *k* ≤ 6
	−25 ≤ *l* ≤ 25
*R_int_*	0.149
*R*_σ_	0.1107
No. of data in refinement	35,347
No. of refined parameters	406
*R*1 [*I* > 2σ(*I*)]	0.052
*wR*2	0.115
Goodness of fit, *S*	0.983
Extreme in residual map/e·Å^−3^	−0.294→0.203
